# Changes in secoiridoids content and chemical characteristics of cultivated and wild Algerian olive oil, in term of fruit maturation

**DOI:** 10.1371/journal.pone.0260182

**Published:** 2021-11-16

**Authors:** Massinissa Faci, Malika Douzane, Mariem Hedjal, Mohamed Seghir Daas, Laëtitia Fougere, Eric Lesellier

**Affiliations:** 1 Department of Biological sciences, Mouloud Mammeri University of Tizi-Ouzou, Tizi-Ouzou, Algeria; 2 Agri-Food Technologies Research Division, National Institute of Agronomic Research of Algeria, Algiers, Algeria; 3 Institute of Organic and Analytical Chemistry, University of Orleans, National Centre for Scientific Research, Orleans, France; National University of Kaohsiung, TAIWAN

## Abstract

Wild varieties in nature are known to be better adapted to climate change and more resistant to arid conditions common in some regions of the world. Oil samples of two cultivated varieties, Chemlal and Lemli, and one sylvestris variety were collected at four different harvesting periods in the semi-arid region of Bouira, Algeria. The aim of this study was to determine the influence of the genetic and maturity factors on the quality indices (acidity, peroxides value, and the parameters K232, K270), fatty acids profile, phenolic composition, and antioxidant activity of monovarietal olive oils. The study showed that early harvest dates of the fruits produced oils richer in pigments and phenolic compounds, with high antioxidant activity registered in both wild and cultivated varieties. Moreover, all oil samples showed high values of secoiridoids exceeding 60–90% of total biophenols, with higher values found in oleaster oils, which are correlated with high resistance to oxidation attacks. UHPLC-DAD and UHPLC-HRMS analyses showed that the secoiridoids composition is dominated by a profile rich in several isomers of oleuropein and ligstroside aglycons, which in turn represent more than 60% of the total secoiridoids in olive and Oleaster oils. Furthermore, chemometric analysis on the data allowed a better appreciation of the sensitivity of the virgin olive oil composition to the changes in genetic and ripening factors. According to the principal component analysis, phenolic and fatty acid profiles were the most important components contributing to the discrimination between olive oil samples.

## Introduction

Fats occupy an important place in dietary intake, are essential for the development and functioning of the human body. The source and quality of fats in food appear to have a major impact on human health [1]. Thus, the Mediterranean region known for its diet rich in olive oil has the lowest incidence in certain diseases described as "disease of the century" worldwide. The fatty acid composition of olive oil differs from other vegetable oils by its high monounsaturated fatty acid composition at the expense of saturated fatty acids. The importance of a high ratio of monounsaturated/saturated fatty acids of the olive oil in daily uses was correlated with a low incidence of coronary and cancer diseases [2]. Other studies [3–5] have demonstrated its effectiveness in reducing the occurrence of many chronic diseases, such as atherosclerosis, rheumatoid arthritis, and nonalcoholic fatty liver disease.

Besides, extra virgin olive oil (EVOO) remains a natural fruit juice resulting from a simple physical extraction without using solvents as required for seed oils, according to International Olive Council regulations (IOC) [6]. This extraction process allows the olive oil to be characterized by a high content of natural bioactive compounds, which confers large bioactivity and authentic sensory quality to this oil. These various nutritional and therapeutic qualities are due to its high content of natural compounds such as vitamins, phytosterols, pigments, and especially phenolic compounds, commonly called “polyphenols” [7]. EVOO contains a wide range of phenolic compounds resulting from secondary plant metabolism, including flavonoids (e.g. apigenin and luteolin), phenolic alcohols (e.g. tyrosol and hydroxytyrosol), phenolic acids (e.g. caffeic acid and vanillic acid), and in particular secoiridoids, which are the most abundant fraction [8, 9].

The health-promoting properties of secoiridoids are recently recognized for their high biological activity against inflammatory, cancer and Alzheimer’s disease; in particular, the secoiridoids oleocanthal and oleacein, which are attracting increasing attention because of their beneficial effects on health and their multiple biological activities [10]. Derived mainly from the de-glucosylation of oleuropein and ligstroside present in olives, the aglycone form of oleuropein and ligstroside are the most abundant secoiridoids in olive oil [11]. These two compounds, defined as specific oleosides of Oleacaea, are sensitive to chemical and enzymatic degradation due to the ester functions carried by the aglycone form [12]. According to Laincer et al. [13], the secoiridoids content is closely related to the genetic characteristics of the olive tree. In other studies [14–16] a relation has been found with the climatic changes, the geographical origin, and the technological process.

Thus, thanks to the diversity of the genetic heritage and the widespread of *Olea europaea* L. cultivation in the world, olive oil production has been marked by a large variation in its lipid and non-lipid composition, due notably to the influence of the multiple factors mentioned previously. The aim of this work is to investigate the influence of the genetic factor and the harvest time on the chemical composition and extent of bioactive compounds of olive oil. The research was performed using separation techniques Ultra High-Pressure liquid chromatography (UHPLC), coupled with Photo-Diode Array detection (DAD) and High-resolution mass spectrometry (HRMS) systems, in order to improve the separation time, ensure high resolution and broad identification of EVOO extracts.

## Materials and methods

### Sample and oil preparation

In order to understand the influence of the genetic resource on the characteristics of virgin olive oils and to give a better assessment of the ripening process contribution to variations of several oil quality parameters; particularly in secoiridoids composition, natural antioxidants content, and fatty acids profile of both wild and cultivated olive varieties. Olive oils were obtained from two endemic Algerian olive cultivars, Chemlal (C) and Limli (L), cultivated in the growing region of Bouira, north-center Algeria. The Oleaster oil was obtained from the wild olive (*Olea europaea* L. subsp. Oleaster (O)) population in the same region. The harvested campaign was carried out during the crop season of 2018, over a period of one month spread through four harvesting dates (H) and spaced out with ten days intervals between them (H1:November 25; H2:December 5; H3:December 15; H4:December 25). The olive samples were manually harvested in random and cleaned carefully by following the sampling method described in the International Olive Council [17].

The olive oil extraction was carried out with a two-phase laboratory oil mill (S.I.O.L. 20240 GHISONACCIA, France). Briefly, the fruits were crushed using a hammer mill equipped with a perforated grid (0.8 mm diameter holes). The homogenous paste was transferred into non-hermetic stainless containers and stirred with rotating pallets for 35 minutes at 25° C; to ensure better coagulation of the oil particles and to limit the oxidation phenomenon. The separation of the phases (oil, wet pomace) was carried out using a vertical centrifuge set at 3000 rpm for 3 min. Finally, the filtered oil samples were stored in opaque glass flasks (protected from light and air oxidation) and 4°C temperature until analysis.

### Maturity index

The estimation of maturity degree was based on the visual observation of the olive mesocarp and epicarp colors, the Maturity Index (MI) were determined by the formula described by International Olive Council [17].

### Quality indices

The progress of triglyceride hydrolysis in olive oil expressed in percentage of free oleic acid (%) was determined following the International Standard Organization procedure [18]. Following the International Olive Council protocols [19, 20], the oxidation level was evaluated by measuring the peroxide value (PV) (Hydroperoxide) and UV spectrophotometer indices (K232 and K270) for oxidation products.

### Fatty acid analysis

The fatty acid profile of the olive oil samples was determined using a Chrompack CP 9002 system equipped with Agilent Cp-Sil 8 CB column (60 m length, 0.25 mm i.d) (5% phenyl+ 95% dimethyl polysiloxane). The fatty acid methyl esters (FAMEs) were prepared by cold transesterification method according to the chromatographic procedure defined by EEC Regulation 2568/91 [21]. Then 0.8 μl was injected under a constant injector temperature of 250°C. The oven temperature increased from 150C° to 200°C by 4°C/min. The nitrogen was used as carrier gas with flow rates of 1ml/min. FAMEs were detected with a flame ionization detector (FID) set at 250°C, and the identification was carried out by comparing their retention times with those of standard reference compounds.

### Pigment determination

The content of chlorophylls and carotenoids was measured following the spectrophotometry method described by Minguez-Mosquera et al. [22]. In brief, 7.5 g of the test sample was dissolved in 25 ml of cyclohexane, and then the mixture was analysed with UV-VIS spectrophotometer set at 670 and 470 nm, which are the maximum absorption for chlorophylls and carotenoids, respectively. The chlorophylls content was calculated as if it were all pheophytin "a", known as the major chlorophylls fraction, using the values of the specific extinction coefficients of this fraction E_0_ = 613. The carotenoids concentration was calculated also according to its main component, lutein, with its specific extinction coefficient E_0_ = 2000. The pigment contents were determined using the following formula:

$$Chlorophyll\;(mg_{pheophytin\;‘a’}/kg)=(A_{670}\times \;10^{6})\;/\;(E_{0}\times \;100\;\times \;d)$$

$$Carotenoid\;(mg_{lutein}/kg)=(A_{470}\times \;10^{6})\;/\;(E_{0}\times \;100\;\times \;d)$$

The results reported are based on oil weight (mg/kg of olive oil). A is the absorbance and d is the thickness of the spectrophotometer cell (1 cm).

### Phenols extraction

The extraction step was carried out according to the International Olive Council method [23], with minor modification. In 20 mL centrifugal tubes, 2 grams of virgin olive oil was mixed with 1ml of syringic acid (used as an internal standard); the latter was prepared in methanol/water mixture (80:20, v/v) with a concentration of 0.015 mg/ml. After 30 seconds of vortex agitation, 5ml of methanol/water (80:20, v/v) were added. Then, the mixture was passed 15 min in ultrasonic bath and 25 min in a centrifuge at 5000 rpm, under 25°C temperature. The supernatant was evaporated with a nitrogen stream until total solvent evaporation, and then the dried polar fraction was reconstituted in 1ml methanol/water (80:20, v/v). The phenolic extracts were filtered through a 0.45μm PVDF filters, and refrigerated until their use.

### UHPLC-DAD and UHPLC-HRMS analysis

The phenol compounds were analysed using a Dionex Ultimate 3000 UHPLC system (Thermo Scientific, USA) equipped with a polar C18 Luna Omega column (100 mm × 2.1 mm id; 1,6 μm particles size), in order to decrease the separation time and ensure a high resolution. A binary gradient of pure water (A) and acetonitrile (B), both previously acidified with 0.1% formic acid, formed the mobile phase. The flow rate was set at 0.4 mL/min and the gradient elution was performed in 16 min as follow: 10% of Bin 0 min, 10% to 35% B for 5min, 35% to 60% B for 2 min, isocratic at 60% for 3 min, 60 to 80% B for 4 min, and 80 to 100% B for 2 min. Four minutes were necessary for the column equilibration between each injection. The oven temperature set at 25°C. Then, 3 μL were automatically deducted from each 1ml of the filtered extract, displayed in different vials. The detection was performed with Diode Array Detector (DAD) in the range of 200–600 nm, and the most phenolic compound were observed at 280 nm wavelength. The same chromatographic conditions were used for the UHPLC–HRMS analysis. The identification of the different phenolic compounds was performed on a maXis UHR-Q-TOF mass spectrometer (Bruker, Bremen, Germany) in negative electrospray ionisation (ESI) mode. The capillary voltage was set at 4.0 kV. The flows of nebulising and drying gas (nitrogen) were set at 2.0 bar and 9.8 L/min, respectively, and the drying gas was heated at 200°C. Analyses were recorded at an acquisition frequency of 2 Hz, and the mass range was set from *m/z* 50 to 1650. Data were processed using DataAnalysis 4.4 (Bruker).

### Phenols quantification method

The determination of the phenolic compound concentrations in the olive oil extracts was carried out according to the International olive council procedure (COI/T.20/Doc. No 29/Rev.1). The syringic acid was added as internal standard during the extraction phase, as explained above. The secoiridoids identified with UHPLC-HRMS were quantified using the sum areas of the corresponding chromatographic peaks recovered by the UHPLC-DAD at 280 nm and the peak area of the internal standard. The secoiridoids amount were presented in mg/kg of tyrosol equivalent, as follow:

$$mg_{Tyrosol\;equivalent}/kg=\frac{\left(\sum A\right)\times 1000\;\times RRF_{syr/tyr}\times W_{syr.\;acid}}{A_{syr.\;acid}\times \;W}$$

ΣA: total of the peak areas of the individual phenols detected at 280 nm;

1000: refers to the factor needed to convert the value into mg/kg;

RRF_syr/tyr_: the multiplication coefficient (RRF_syr/tyr_ = 4.74) to express the result in tyrosol equivalent;

A _syr. Acid_: the internal standard (Syringic acid) area detected at 280 nm;

W _syr.acid_: the weight of the syringic acid (mg) added as internal standard.

W: the weight (g) of the tested olive oil;

### Determination of total phenol

The total phenol contents were measured using the modification of the procedure described by Singleton and Rossi [24], based on the reduction of phosphomolybdic-phosphotungstic acid. In a simple 96-well microplate, 20 ul of each phenolic extract were transferred, with 20 ul of Folin ciocalteau reagent. After 5 min waiting, the coloration of the reaction was activated by 30 ul of the 20% aqueous sodium carbonate solution. Finally, the mixture was diluted with 140 ul of distilled water. Therefore, the same steps were carried out to prepare the Gallic acid calibration curve, including concentrations range of 0.05, 0.1, 0.2, 0.3, 0.4 and 0.5 mg/ml. all the preparations were read under a microplate spectrophotometer (Thermo scientific multiskan, USA) at 760 nm, after 25°C incubation for 2 hours in the dark. Using the Gallic acid calibration curve, the total phenol content was quantified in mg Gallic acid/kg oil.

### DPPH antiradical reaction

The DPPH antiradical capacity was estimated using the method reported by Tepe et al. [25]. 10 μl of each olive oil extract and calibration solution of Trolox (1, 0.8, 0.5, 0.25, 0.1 and 0.05 mg/ml) were complemented with 190 μl of a DPPH solution (2,2-diphenyl-1-picrylhydrazyl) concentrated at 0.15%, in methanol. After 30 seconds stirring, the mixtures were incubated for 30 min at 25°C. During this step, a change from dark violet to pale yellow color was observed, due to the reduction of free radicals present in DPPH form by the antioxidant power of the phenolic compounds. The absorbance was read with a microplate spectrophotometer at 516 nm. The values of scavenging activities were expressed as mg trolox equivalents/ml (mg TE/ml).


$$\%Inhibition=\left\{1-\left(A_{sample}/A_{control}\right)\right\}\times 100$$

*A*_control_ is control test that was measured as the absorbance of DPPH without sample addition.

### Statistical and chemometric analysis

The statistical analyses were performed with Minitab 19 (Minitab software, USA). One-way variance analysis test (ANOVA) was carried out on the oil data, followed by Tukey post-hoc test, with significance set at P<0.05. For improved discrimination and better visualization of data variation, principal component analysis (PCA) was achieved on the most important oil parameters.

## Results and discussions

### Maturity index

During the ripening process, photosynthetic activity decreases progressively in the olive fruit [26], and expressed by different range of color going from green, purple to black, which allows the ripening process to be evaluated through the maturity index [27, 28]. At the first harvest date (H1) of November 25, the olives were found with low maturity indices of 2.30, 2.48 and 1.33 in the cv. Chemlal, cv. Limli and Oleaster, respectively (Table 1).

**Table 1:** Physico-chemical proprieties of EVOO samples.

Varieties	Harvest date	Maturity index	Acidity (%)	Peroxide value (MeqO_2_/Kg)	K232 (nm)	K270 (nm)
**Chemlal**	H1	2.30 ± 0.20 ^a^^;^^x^	0.34 ± 0.06 ^a^^;^^w^^x^	10.43 ± 1.04 ^b^^;^^w^	1.18 ± 0.01 ^b^^;^^v^	0.24 ± 0.00 ^a^^b^^;^^v^
**Chemlal**	H2	3.48 ± 0.27 ^a^^;^^w^	0.24 ± 0.02 ^a^^;^^x^	11.36 ± 0.99 ^b^^;^^v^^w^	0.86 ± 0.05 ^b^^;^^v^	0.19 ± 0.01 ^a^^b^^;^^v^
**Chemlal**	H3	4.74 ± 0.48 ^a^^;^^v^	0.40 ± 0.13 ^a^^;^^v^^w^	12.19 ± 0.30 ^b^^;^^v^^w^	0.78 ± 0.02 ^b^^;^^v^	0.15 ± 0.01 ^a^^b^^;^^v^
**Chemlal**	H4	5.33 ± 0.33 ^a^^;^^v^	0.53 ± 0.14 ^a^^;^^v^	9.29 ± 0.96 ^b^^;^^v^	1.00 ± 0.06 ^b^^;^^v^	0.15 ± 0.00 ^a^^b^^;^^v^
**Limli**	H1	2.44 ± 0.16 ^b^^;^^x^	0.36 ± 0.07 ^a^^;^^w^^x^	7.79 ± 0.83 ^a^^;^^w^	1.54 ± 0.08 ^a^^;^^v^	0.23 ± 0.00 ^a^^;^^v^
**Limli**	H2	2.87± 0.13 ^b^^;^^w^	0.36 ± 0.03 ^a^^;^^x^	10.54 ± 0.40 ^a^^;^^v^^w^	1.04 ± 0.04 ^a^^;^^v^	0.17 ± 0.00 ^a^^;^^v^
**Limli**	H3	3.87 ± 0.38 ^b^^;^^v^	0.45 ± 0.00 ^a^^;^^v^^w^	13.58 ± 0.07 ^a^^;^^v^^w^	1.11 ± 0.14 ^a^^;^^v^	0.19 ± 0.00 ^a^^;^^v^
**Limli**	H4	4.35 ± 0.39 ^b^^;^^v^	0.57 ± 0.00 ^a^^;^^v^	15.75 ± 1.44 ^a^^;^^v^	1.37 ± 0.00 ^a^^;^^v^	0.22 ± 0.00 ^a^^;^^v^
**Oleaster**	H1	1.33 ± 0.09 ^a^^b^^;^^x^	0.40 ± 0.00 ^a^^;^^w^^x^	13.50 ± 0.71 ^a^^;^^w^	0.94 ± 0.16 ^b^^;^^v^	0.15 ± 0.01 ^b^^;^^v^
**Oleaster**	H2	2.36 ± 0.22 ^a^^b^^;^^w^	0.33 ± 0.11^a^^;^^x^	15.00 ± 2.12 ^a^^;^^v^^w^	0.95 ± 0.06 ^b^^;^^v^	0.19 ± 0.02 ^b^^;^^v^
**Oleaster**	H3	2.90 ± 0.26 ^a^^b^^;^^v^	0.40 ± 0.07 ^a^^;^^v^^w^	13.50 ± 0.71 ^a^^;^^v^^w^	0.91 ± 0.10 ^b^^;^^v^	0.14 ± 0.01 ^b^^;^^v^
**Oleaster**	H4	3.65 ± 0.41 ^a^^b^^;^^v^	0.38 ± 0.04 ^a^^;^^v^	15.50 ± 0.00 ^a^^;^^v^	0.86 ± 0.05 ^b^^;^^v^	0.12 ± 0.00 ^b^^;^^v^

Mean± standard deviation. Different letters in the same row indicate significant differences (p <0.05),

^a-c^ refers the varietal effect and

^v-x^ to the Harvest time effect.

**Fig 1:**
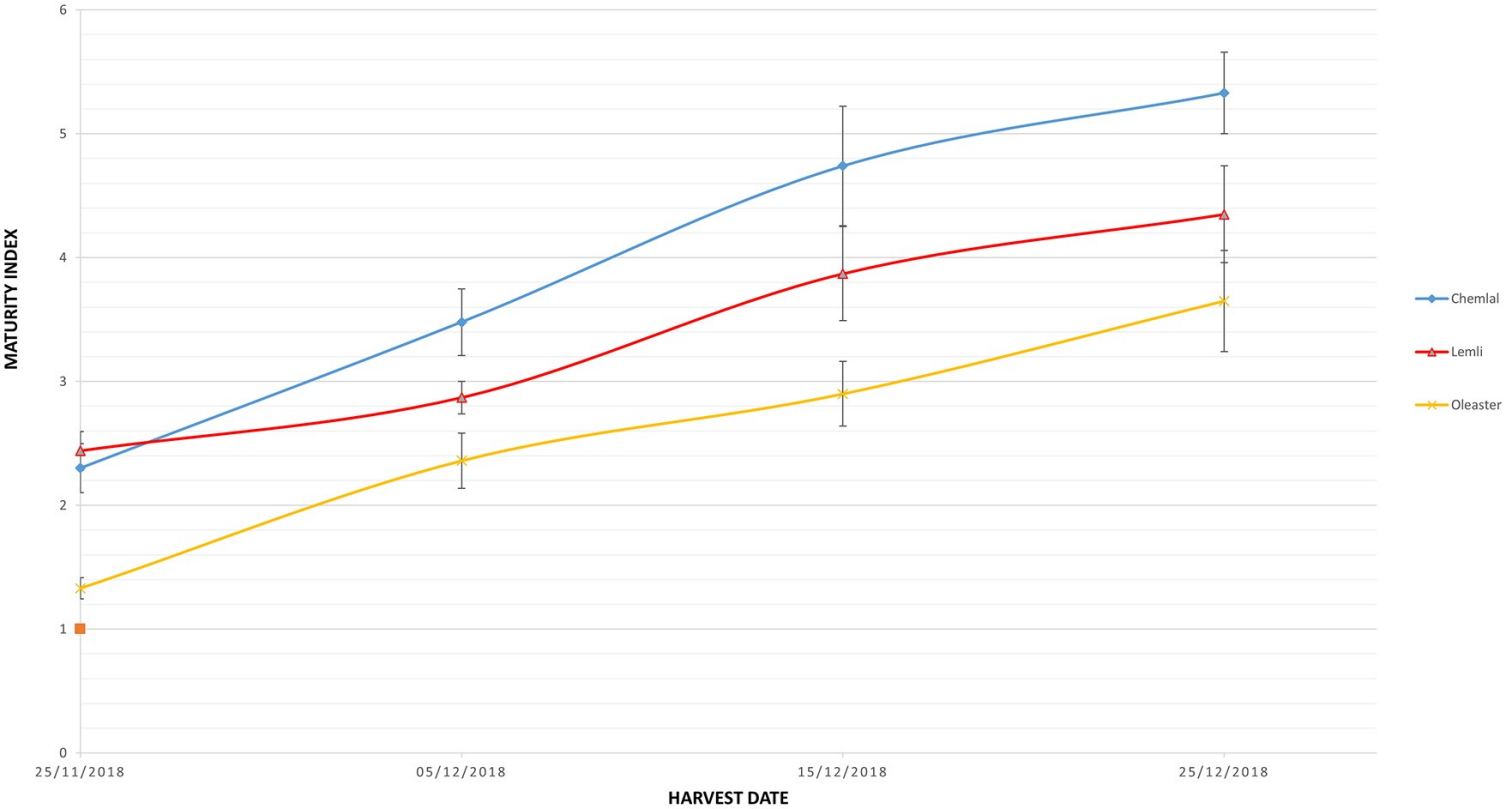
Changes in maturity index during the different harvest dates.

Therefore, wild olives were characterized by the lowest maturity values ranging from 1.33 to 3.65 through harvest periods H1 to H4. This difference was probably due to the late entry of wild fruits in maturation compared to the cultivated varieties (Fig 1).

On another side, a fast ripening process was observed in the olives of the cv. Chemlal, which attained the advanced ripening level of 5.33 (Black) at the last harvest (H4). Furthermore, data analysis showed significant differences (P≤0.05) between the varieties and confirm the influence of the genetic factor on the olive maturity process, as Baccouri et al. [8] reported in their study.

### Quality parameters

The majority of olive oil collected from different sampling conditions (olive Variety and Maturation) were found under the limit established by the IOC (IOC/T.15/NC No. 3/Rev 8), for free acidity (≤ 0.8%), peroxide value (≤ 20 meqO_2_/kga), K232 (≤ 2.50) and K270 (≤ 0.22) (Table 1). Thus, all oil samples were categorized as EVOO.

ANOVA analysis showed significant (P≤ 0.05) influences of varietal component on the oxidative parameters of the olive oil. Indeed, the Oleaster oil presented low values of UV spectrophotometric, that ranging from 0.86 to 0.95 for primary oxidation (K232) and from 0.12 to 0.19 for secondary oxidation product (K270), and which found higher in Chemlal and Limli cultivars. Bouarroudj et al. [29] reported comparable results on Oleaster oils. The data listed in Table 1 showed no significant variation (P≥ 0.05) in the changes in K232 and K270 values during the progression of maturity. Free acidity (FFA) percentage showed a slight but significant increase (P = 0.00) during the ripening process, especially in Chemlal and Limli cultivars, but remains below the limit of 0.8% (for EVOO) even in the advanced stages of maturity. The rise of FFA content was related to the increase activity of lipolytic enzyme during the olive-ripening phase [8].

### Fatty acids composition

The fatty acids (FA) constitute the major fraction of olive oil and its composition is considered as a marker for the classification and the determination of the varietal and geographical origin of olive oil [14, 30]. The most abundant FA found in all EVOO samples was oleic acid, as observed in other researches [8, 14] (Table 2).

**Table 2:** Fatty acids composition of olive oil samples (%).

Varieties	Harvest date	C16: 0	C16: 1	C17: 0	C18: 0	C18: 1	C18: 2	C18: 3	C20: 0	C20: 1	C22: 0	C18:1/ C18:2	MUFA/ SFA	MUFA/ PUFA
**Chemlal**	H1	14.08 ^a^^;^^v^	1.66 ^a^^;^^v^	0.12 ^b^^;^^v^	1.90 ^a^^b^^;^^v^	72.55 ^a^^b^^;^^v^	8.23 ^a^^b^^;^^v^	0.60 ^b^^;^^v^^w^	0.34 ^a^^;^^v^^w^	0.31 ^a^^;^^w^	0.12 ^a^^;^^v^^w^	8.82 ^a^^b^^;^^v^	4.50 ^a^^;^^v^	8.43 ^a^^b^^;^^v^
**Chemlal**	H2	17.19 ^a^^;^^v^	2.06 ^a^^;^^v^	0.09 ^b^^;^^v^	1.76 ^a^^b^^;^^v^	68.31 ^a^^b^^;^^v^	9.35 ^a^^b^^;^^v^	0.33 ^b^^;^^w^	0.33 ^a^^;^^w^	0.34 ^a^^;^^v^^w^	0.00 ^a^^;^^w^	7.31 ^a^^b^^;^^v^	3.65 ^a^^;^^v^	7.30 ^a^^b^^;^^v^
**Chemlal**	H3	12.50 ^a^^;^^v^^w^	1.55 ^a^^;^^v^	0.10 ^b^^;^^v^	1.92 ^a^^b^^;^^v^	71.43 ^a^^b^^;^^v^	11.51 ^a^^b^^;^^v^	0.55 ^b^^;^^v^^w^	0.33 ^a^^;^^w^	0.30 ^a^^;^^v^^w^	0.12 ^a^^;^^v^^w^	6.21 ^a^^b^^;^^v^	4.90 ^a^^;^^v^	6.07 ^a^^b^^;^^v^
**Chemlal**	H4	13.47 ^a^^;^^w^	1.69 ^a^^;^^v^	0.12 ^b^^;^^v^	1.85 ^a^^b^^;^^v^	72.94 ^a^^b^^;^^v^	8.34 ^a^^b^^;^^v^	0.60 ^b^^;^^v^	0.35 ^a^^;^^v^	0.36 ^a^^;^^v^	0.13 ^a^^;^^v^	8.75 ^a^^b^^;^^v^	4.71 ^a^^;^^v^	8.38 ^a^^b^^;^^v^
**Limli**	H1	18.30 ^a^^;^^v^	1.20 ^c^^;^^v^	0.25 ^a^^;^^v^	2.61 ^a^^;^^v^	64.71 ^b^^;^^v^	11.48 ^a^^;^^v^	0.62 ^a^^b^^;^^v^^w^	0.38 ^a^^;^^v^^w^	0.28 ^a^^;^^w^	0.12 ^a^^;^^v^^w^	5.64 ^b^^;^^v^	3.06 ^b^^;^^v^	5.47 ^b^^;^^v^
**Limli**	H2	16.60 ^a^^;^^v^	1.10 ^c^^;^^v^	0.23 ^a^^;^^v^	2.23 ^a^^;^^v^	68.11 ^b^^;^^v^	10.21 ^a^^;^^v^	0.36 ^a^^b^^;^^w^	0.36 ^a^^;^^w^	0.32 ^a^^;^^v^^w^	0.12 ^a^^;^^w^	6.67 ^b^^;^^v^	3.56 ^b^^;^^v^	6.57 ^b^^;^^v^
**Limli**	H3	14.76 ^a^^;^^v^^w^	1.14 ^c^^;^^v^	0.25 ^a^^;^^v^	2.27 ^a^^;^^v^	70.39 ^b^^;^^v^	9.67 ^a^^;^^v^	0.69 ^a^^b^^;^^v^^w^	0.34 ^a^^;^^w^	0.32 ^a^^;^^v^^w^	0.12 ^a^^;^^v^^w^	7.28 ^b^^;^^v^	4.05 ^b^^;^^v^	6.93 ^b^^;^^v^
**Limli**	H4	12.47 ^a^^;^^w^	0.99 ^c^^;^^v^	0.23 ^a^^;^^v^	2.14 ^a^^;^^v^	72.45 ^b^^;^^v^	10.00 ^a^^;^^v^	0.76 ^a^^b^^;^^v^	0.39 ^a^^;^^v^	0.38 ^a^^;^^v^	0.14 ^a^^;^^v^	7.25 ^b^^;^^v^	3.25 ^b^^;^^v^	6.86 ^b^^;^^v^
**Oleaster**	H1	14.40 ^a^^;^^v^	1.56 ^b^^;^^v^	0.09 ^b^^;^^v^	2.06 ^b^^;^^v^	72.57 ^a^^;^^v^	7.79 ^b^^;^^v^	0.76 ^a^^;^^v^^w^	0.35 ^a^^;^^v^^w^	0.26 ^b^^;^^w^	0.12 ^a^^;^^v^^w^	9.32 ^a^^;^^v^	4.28 ^a^^;^^v^	8.53 ^a^^;^^v^
**Oleaster**	H2	13.55 ^a^^;^^v^	1.35 ^b^^;^^v^	0.10 ^b^^;^^v^	2.08 ^b^^;^^v^	73.30 ^a^^;^^v^	8.20 ^b^^;^^v^	0.72 ^a^^;^^w^	0.33 ^a^^;^^w^	0.23 ^b^^;^^v^^w^	0.10 ^a^^;^^w^	8.94 ^a^^;^^v^	4.56 ^a^^;^^v^	8.26 ^a^^;^^v^
**Oleaster**	H3	14.69 ^a^^;^^v^^w^	1.66 ^b^^;^^v^	0.09 ^b^^;^^v^	1.98 ^b^^;^^v^	71.18 ^a^^;^^v^	8.96 ^b^^;^^v^	0.69 ^a^^;^^v^^w^	0.34 ^a^^;^^w^	0.26 ^b^^;^^v^^w^	0.11 ^a^^;^^v^^w^	7.94 ^a^^;^^v^	4.16 ^a^^;^^v^	7.41 ^a^^;^^v^
**Oleaster**	H4	13.16 ^a^^;^^w^	1.15 ^b^^;^^v^	0.11 ^b^^;^^v^	0.47 ^b^^;^^v^	74.18 ^a^^;^^v^	9.05 ^b^^;^^v^	0.77 ^a^^;^^v^	0.45 ^a^^;^^v^	0.31 ^b^^;^^v^	0.21 ^a^^;^^v^	8.20 ^a^^;^^v^	5.18 ^a^^;^^v^	7.60 ^a^^;^^v^

The amount of the different fatty acids were determined in percentage. C16:0, Palmitic; C18:0, Stearic; C16:1, Palmitoleic; C17:0, margaric; C18:1, Oleic; C18:2, Linoleic; C18:3, Linolenic; C20:0, arachidic; C20:1, gadoleic; C22:0, behenic acids. MUFA/PUFA, Monounsaturated fatty acids/ Poly-unsaturated fatty acids ratio; UFA/SFA; Unsaturated fatty acids/ Saturated fatty acids ratio. Different letters in the same row indicate significant differences (p <0.05),

^a-c^ refers the varietal effect and

^v-x^ to the Harvest time effect.

During the olive maturation, its percentage fluctuates between 72.55 and 72.94% in the cv. Chemlal, between 64.71 and 72.45% in the cv. Limli and from 72.57 to 74.18% in the Oleaster oil; the changes in oleic content were found with significant differences among the studied varieties (P = 0.007) and no significant in terms of maturity progression (P≥ 0.05). Those results are quite similar to other data carried on the Algerian varieties [29, 31]. However, in the course of fruit ripening, the maximum values of oleic acid were observed at the last harvest date (H4) in all olive varieties; This behavior may indicate that the oleic acid synthesis continues even at the late stages of the ripening process, as observed by others researchers [32, 33].

As reported by Dessouky et al. [34] and Rondanini et al. [1], the percentage of the main saturated fatty acid -palmitic acid in the EVOO samples, showed different behavior between varieties during fruit maturation. In fact, a high level of palmitic acid content was observed at H2 in cv. Chemlal and H1 in cv. Limli; and was followed by an important decrease at advanced ripening stages. In comparison, the Oleaster oils presented no clear variation (P≥0.05) in this fatty acid during fruit maturation. Numerous studies [8, 28], were related the drop in palmitic acid ratio during the advanced ripening stages to the dilution effect, caused by stability in palmitic acid percent and the increased level of other fatty acids.

The ratio of oleic/linoleic, MUFA/SFA (mono-unsaturated fatty acid/saturated fatty acid) and MUFA/PUFA (poly-unsaturated fatty acid) were related with both nutritional aspect and oxidative stability of olive oil [32]. During harvest dates H1, H2 and H3, the Oleaster oil showed a highest oleic/linoleic ratio of 9.32, 8.99 and 7.94 compare to the composition Chemlal and Limli oil at the same periods, therefore, the MUFA/PUFA parameter showed a same and significant difference (P≤0.05) between the varieties. Those characteristics, can procure Oleaster oil more stability and longer shelf life during the storage [32, 35]. Despite the fact that no significant differences (P≥0.05) showed in term of olive ripens, in general, a slight decrease can be noticed in both C18:1/C18:2 and MUFA/PUFA parameters at the last harvest date, compare to the first harvest (H1) in Chemlal and Oleaster oils; and third harvest (H3) in Limli oil. For Yorulmaz et al. [33], this decrease was possibly due to the transformation oleic into linoleic acid caused by the activity of oleate desaturase enzyme. In addition, the values of MUFA/SFA ratio reached their maximum at advanced maturation stages, with levels of 4.90 and 4.05 at H3 in oils provided from Chemlal and Limli cultivars, and 5.18 at H4 in Oleaster oil. This behavior can be associated to low levels of palmitic acids at the end of maturation. For Beltrán et al., [32], this relationship is due to the increasing activity of *β*-ketoacyl-ACP synthase II (KAS II) enzyme, which induces the chain elongation of palmitoyl-ACP to stearoyl-ACP.

### Pigment content

During the olive ripening process, important changes occur in the pigment content, which constantly affect the color, flavor and antioxidant capacity of the extracted olive oil [36]. In the early olive piking, a high level of chlorophylls and carotenoids content was observed in all oil samples (Table 3), and was particularly pronounced in the cv. Limli, with amounts of 11.61 and 5.71 mg/kg found in both pigments, respectively.

**Table 3:** Pigment, total phenol and antioxidant proprieties of EVOO samples.

Varieties	Harvest date	Chlorophylls (mg/kg)	Carotenoids (mg/kg)	Total phenol (mg CAE/kg)	DPPH (%)	DPPH (mg TE/ml)
**Chemlal**	H1	9.45 ± 1.96 ^a^^;^^v^	3.58 ± 0.91^a^^;^^v^	183.57 ± 1.20 ^b^^;^^v^	79.84 ± 0.84 ^b^^;^^v^	0.448 ± 0.005 ^b^^;^^v^
**Chemlal**	H2	7.29 ± 0.00 ^a^^;^^w^	2.38 ± 1.08 ^a^^;^^w^	170.78 ± 15.32 ^b^^;^^v^	79.56 ± 2.07 ^b^^;^^v^^w^	0.446 ± 0.001 ^b^^;^^v^^w^
**Chemlal**	H3	7.50 ± 0.00 ^a^^;^^x^	2.56 ± 1.34 ^a^^;^^w^	150.11 ± 5.54 ^b^^;^^v^	36.06 ± 3.86 ^b^^;^^x^	0.207 ± 0.002 ^b^^;^^x^
**Chemlal**	H4	6.65 ± 0.24 ^a^^;^^x^	2.66 ± 0.51 ^a^^;^^w^	199.02 ± 4.21 ^b^^;^^v^	24.17 ± 0.96 ^b^^;^^x^	0.141 ± 0.005 ^b^^;^^x^
**Limli**	H1	11.61 ± 1.65 ^a^^;^^v^	5.71 ± 0.37 ^a^^b^^;^^v^	187.70 ± 20.4 ^a^^;^^v^	61.95 ± 1.53 ^c^^;^^v^	0.349 ± 0.008 ^c^^;^^v^
**Limli**	H2	7.52 ± 0.28 ^a^^;^^w^	3.85 ± 1.49 ^a^^b^^;^^w^	115.44 ± 8.61 ^a^^;^^v^	32.78 ± 0.94 ^c^^;^^v^^w^	0.189 ± 0.005 ^c^^;^^v^^w^
**Limli**	H3	5.13 ± 1.50 ^a^^;^^x^	3.17 ± 1.00 ^a^^b^^;^^w^	136.00 ± 16.29 ^a^^;^^v^	10.72 ± 1.02 ^c^^;^^x^	0.067 ± 0.006 ^c^^;^^x^
**Limli**	H4	3.74 ± 0.21 ^a^^;^^x^	2.85 ± 0.23 ^a^^b^^;^^w^	114.00 ± 21.80 ^a^^;^^v^	18.39 ± 2.11 ^c^^;^^x^	0.109 ± 0.001 ^c^^;^^x^
**Oleaster**	H1	9.95 ± 2.28 ^a^^;^^v^	4.34 ± 0.21 ^b^^;^^v^	208.20 ± 38.40 ^a^^;^^v^	89.93 ± 8.73 ^a^^;^^v^	0.503 ± 0.005 ^a^^;^^v^
**Oleaster**	H2	5.38 ± 1.21 ^a^^;^^w^	2.42 ± 0.16 ^b^^;^^w^	181.55 ± 10.58 ^a^^;^^v^	77.85 ± 2.08 ^a^^;^^v^^w^	0.437 ± 0.001 ^a^^;^^v^^w^
**Oleaster**	H3	4.20 ± 1.67 ^a^^;^^x^	1.82 ± 0.07 ^b^^;^^w^	167.29 ± 5.76 ^a^^;^^v^	60.32 ± 7.59 ^a^^;^^x^	0.340 ± 0.004 ^a^^;^^x^
**Oleaster**	H4	4.12 ± 1.14 ^a^^;^^x^	2.09 ± 0.22 ^b^^;^^w^	226.98 ± 15.29 ^a^^;^^v^	74.66 ± 0.86 ^a^^;^^x^	0.419 ± 0.005 ^a^^;^^x^

Mean± standard deviation. Different letters in the same row indicate significant differences (p <0.05),

^a-c^ refers the varietal effect and

^v-x^ to the Harvest time effect.

Numerous studies have related this small variation to influences of the varietal component on pigment production [34]. During the olive maturation, olive oils showed a significant (P≤0.05) and linear decrease in pigment content, by a loss of more than 50% of total chlorophylls and carotenoids, registered at the end of December in Limli and Oleaster oils. This decrease was possibly due to the transformation of chlorophyll (a) and (b) to pheophytin (a) and (b), which provokes a change in olive oil coloration from green to yellow; and affects directly the antioxidant capacities of both pigments and particularly the pro-oxidant activity of the chlorophylls [7, 8].

### Colorimetric total phenol

Total phenol content (TPC) estimated by the Folin-Ciocalteu reagent is known as a routine technique for assessing the main and non-specified phenolic compounds of the olive oil. The amount of total phenol listed in the Table 3, showed a non-clear trend during the ripening process (P≥0.05). Nevertheless, linear decreases can be noticed through the maturation periods H1 to H3 in all varieties; which fell respectively from 183.57 to 150.11 mg CAE/kg; 187.70 to 136.00 mg CAE/kg and from208.20 to 167.29 in the Chemlal, Limli and Oleaster EVOO samples. This behavior is in agreement with the finding reported in study of Zaringhalami et al. [35]. However, in Chemlal and Oleaster oil samples; this decrease was followed by an important rise of the phenol content at last harvest date (H4). This augmentation in TPC can be the result of accumulation of other phenols such anthocyanins at the advanced ripening stage [33], which also have capacity to interact with Folin-Ciocalteu reagent.

Variance analysis on the oils data revealed a high significant (P≤0.001) influence of varietal trait on the polyphenol content. Besides, the averages of polyphenol content was found particularly high in the Oleaster oils (mean of TPC from H1 to H4: 196.00 mg CAE/kg) compare to the cultivated varieties, Chemlal and Limli. Other works done on these varieties [29] are in conformity with our results on the total phenol content. According to Boucheffa et al. [37], the richness of wild olive oils in the phenols content is associated with increased resistance to hydric stress condition of its trees. Indeed, water stress enhance the activity Phenylalanine-Lyase enzyme, known as responsible for the phenylpropanoids biosynthesis in the plant [38].

### UHPLC-HRMS/MS determination of secoiridoids

**Fig 2:**
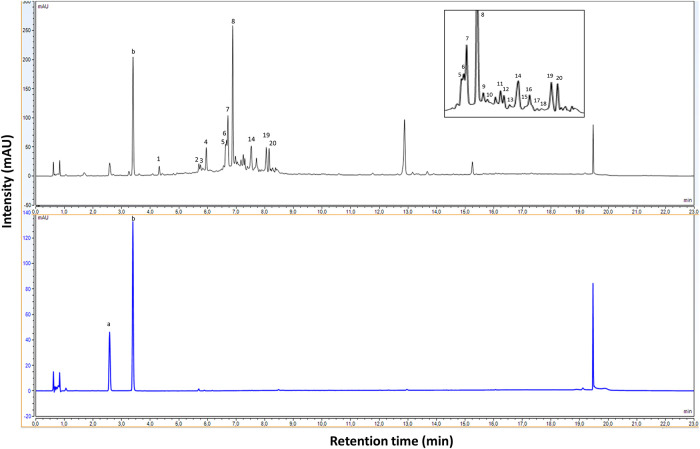
UHPLC chromatographs of olive oil phenolic extract and phenol standard peaks (a: Tyrosol, b: Syringic acid) detected at 280 nm.

Liquid chromatography-mass spectrometry technique is largely used in characterization and structural determination of the EVOO phenolic compounds, and especially the negative ionization mode (ESI) due to its high sensitivity [9]. UHPLC- DAD (Fig 2) and ESI-HRMS investigation into the phenolic profile of oils extracted from Chemlal, Limli and Oleaster varieties have detected 20 secoiridoids compounds (Table 4).

**Table 4:** HRMS-ESI data of the identified secoiridoids compounds in the EVOO samples.

Peak number	Secoiridoid compounds	RT [min]	Ion Formula	Meas. m/z	m/z	err [ppm]
**1**	Elenolic acid	4.43	C11H13O6	241.071	241.072	0.7
**2**	Oleuropein aglycon_1_	5.71	C19H21O8	377.125	377.124	-0.9
**3**	Oleuropein aglycon_2_	5.77	C19H21O8	377.125	377.124	-0.9
**4**	Oleuropein aglycon_3_	5.97	C19H21O8	377.125	377.124	-0.9
**5**	Oleacein	6.57	C17H19O6	319.119	319.119	-0.7
**6**	Ligstroside aglycon_1_	6.65	C19H21O7	361.130	361.129	-1.1
**7**	Ligstroside aglycon_2_	6.68	C19H21O7	361.130	361.129	-1.1
**8**	Ligstroside aglycon_3_	6.91	C19H21O7	361.130	361.129	-1.1
**9**	Oleuropein aglycon_4_	7.00	C19H21O8	377.124	377.124	-0.5
**10**	Dimethyl acetal of oleacein	7.12	C19H25O7	365.161	365.161	-0.8
**11**	Oleuropein aglycon_5_	7.23	C19H21O8	377.124	377.124	-0.1
**12**	Ligstroside aglycon_4_	7.27	C19H21O7	361.130	361.129	-0.4
**13**	Methyl oleuropein aglycon_1_	7.44	C20H23O8	391.140	391.140	-0.8
**14**	Oleuropein aglycon_6_	7.54	C19H21O8	377.124	377.124	-0.8
**15**	Oleuropein aglycon_7_	7.68	C19H21O8	377.124	377.124	-0.8
**16**	Ligstroside aglycon_5_	7.74	C19H21O7	361.130	361.129	-0.6
**17**	Dimethyl acetal of oleocanthal	7.79	C19H25O6	349.166	349.166	-0.3
**18**	Methyl oleuropein aglycon_2_	7.93	C20H23O8	391.140	391.140	-0.4
**19**	Ligstroside aglycon_6_	8.09	C19H21O7	361.130	361.129	-0.7
**20**	Methyl oleuropein aglycon_3_	8.20	C20H23O8	391.140	391.140	-0.9

The identification of the individual phenols was carried out by HPLC-MS. The relative retention time is calculated by comparing it to the retention time of syringic acid.

The secoiridoids composition was characterized mainly by several isomers from oleuropein aglycone (3,4-DHPEA-EA), ligstroside aglycone (p-HPEA-EA), methyl oleuropein aglycone, but also by different derivatives of oleocanthal, oleacein, and elenolic acid. In line with the results obtained by Dierkes et al. [39], at least seven isomers of oleuropein aglycone and six isomers of ligstroside aglycone have been identified. According to the work of Cardoso et al. [40], several isomers of oleuropein and ligstroside are also present in oil mill wastewater and have explained this finding by different glycosylation positions found between the glucose unit and hydroxytyrosol or tyrosol, respectively. Other studies conducted on phenolic extracts of olive oil revealed the presence of diastereoisomers/geometrical isomers and stable enolic/dienolic tautomers that provide indirect structural confirmation for some isomers of the oleuropein aglycon [41, 42]. The analysis of the secoiridoids fraction (Table 4) indicated the presence of acetal form of oleocanthal and oleacein in the EVOO extracts. The latter are known for their high bioactivities and nutritional interest [10]. The formation of hemiacetal/acetal and derivatives occurs during the extraction phase due to the interaction of the protic solvent (water, methanol) with the two secoiridoids, as mentioned by Celano et al. [9]. According to de Medina et al. [43], LC separation with acidified methanol is the potential promoter of acetals and hemiacetals formation. Thus, these fractions have been limited by the use of acetonitrile as the mobile phase.

### Secoiridoids content

Secoiroids constitute the major fraction of the phenolic compounds in EVOO [9, 44]. Their contribution has been linked to the sensory attributes [39] and to several therapeutic properties of olive oil [10]. The data listed in Table 5, revealed that secoiridoids amount represents 60 to 90% of the total individual biophenols of EVOO samples.

**Table 5:** Secoiridoids and total biophenols content (mg _Tyrosol equivalent_/kg) of EVOO samples.

Varieties	Harvest date	Elenolic acid	Oleuropein aglycon and isomers	Ligstroside aglycon and isomers	Oleacein and derivative	Oleocanthal and derivative	Methyl oleuropein aglycon	Total secoiridoids	Total Biophenols HPLC
**Chemlal**	H1	21.88 ± 0.16 ^b^^;^^v^	31.07 ± 3.27 ^b^^;^^v^	79.41 ± 7.68 ^b^^;^^v^	3.50 ± 0.72 ^a^^;^^v^	1.46 ± 0.99 ^b^^;^^v^	42.87 ± 2.8 ^b^^;^^v^	180.21 ± 14.96 ^b^^;^^v^	199.54 ± 4.47 ^b^^;^^v^
**Chemlal**	H2	8.77 ± 0.85 ^b^^;^^w^	30.82 ± 6.95 ^b^^;^^v^	110.20 ± 23.60 ^b^^;^^v^	4.68 ± 1.41 ^a^^;^^v^	4.68 ± 0.44 ^b^^;^^v^	10.03 ± 7.31 ^b^^;^^v^	169.20 ± 26.90 ^b^^;^^v^^w^	188.24 ± 23.32 ^b^^;^^v^
**Chemlal**	H3	4.57 ± 0.96 ^b^^;^^w^	12.18 ± 1.08 ^b^^;^^v^	23.14 ± 5.25 ^b^^;^^v^	0.57 ± 0.08 ^a^^;^^w^	2.33 ± 0.10 ^b^^;^^v^	22.72 ± 2.19 ^b^^;^^v^	65.50 ± 8.02 ^b^^;^^w^^x^	95.91 ± 8.49 ^b^^;^^v^^w^
**Chemlal**	H4	4.77 ± 0.60 ^b^^;^^w^	11.63 ± 1.22 ^b^^;^^v^	15.13 ± 2.00 ^b^^;^^v^	0.33 ± 0.04 ^a^^;^^w^	2.60 ± 0.17 ^b^^;^^v^	18.94 ± 2.34 ^b^^;^^v^	53.41 ± 6.37 ^b^^;^^x^	68.08 ± 1.32 ^b^^;^^w^
**Limli**	H1	37.32 ± 1.93 ^a^^;^^v^	19.92 ± 1.58 ^b^^;^^v^	50.38 ± 4.98 ^c^^;^^v^	1.96 ± 0.48 ^a^^;^^v^	12.15 ± 1.01 ^a^^;^^v^	50.66 ± 4.58 ^a^^;^^v^	172.39 ± 14.38 ^b^^;^^v^	190.17 ± 10.88 ^b^^;^^v^
**Limli**	H2	15.25 ± 1.63 ^a^^;^^w^	16.44 ± 1.18 ^b^^;^^v^	19.56 ± 1.85 ^c^^;^^v^	1.80 ± 0.11 ^a^^;^^v^	10.51 ± 1.04 ^a^^;^^v^	58.93 ± 5.79 ^a^^;^^v^	122.49 ± 11.37 ^b^^;^^v^^w^	159.88 ± 15.27 ^b^^;^^v^
**Limli**	H3	16.64 ± 0.17 ^a^^;^^w^	16.45 ± 0.63 ^b^^;^^v^	20.27 ± 0.94 ^c^^;^^v^	1.64 ± 0.02 ^a^^;^^w^	10.96 ± 0.13 ^a^^;^^v^	57.27 ± 0.56 ^a^^;^^v^	123.23 ± 1.59 ^b^^;^^w^^x^	161.83 ± 1.55 ^b^^;^^w^^v^
**Limli**	H4	6.99 ± 0.97 ^a^^;^^w^	15.67 ± 1.22 ^b^^;^^v^	17.22 ± 1.86 ^c^^;^^v^	1.32 ± 0.09 ^a^^;^^w^	8.93 ± 0.62 ^a^^;^^v^	40.99 ± 3.74 ^a^^;^^v^	91.13 ± 8.42 ^b^^;^^x^	113.09 ± 4.66 ^b^^;^^w^
**Oleaster**	H1	8.10 ± 0.37 ^b^^;^^v^	37.52 ± 8.20 ^a^^;^^v^	124.21 ± 10.51 ^a^^;^^v^	3.80 ± 0.62 ^a^^;^^v^	0.33 ± 0.57 ^c^^;^^v^	11.11 ± 0.89 ^c^^;^^v^	185.10 ± 19.90 ^a^^;^^v^	205.23 ± 15.72 ^a^^;^^v^
**Oleaster**	H2	12.25 ± 3.41 ^b^^;^^w^	28.88 ± 4.95 ^a^^;^^v^	124.10 ± 22.30 ^a^^;^^v^	2.14 ± 0.46 ^a^^;^^v^	0.00 ± 0.00 ^c^^;^^v^	12.29 ± 1.68 ^c^^;^^v^	179.60 ± 31.50 ^a^^;^^v^^w^	217.90 ± 34.00 ^a^^;^^v^
**Oleaster**	H3	12.17 ± 0.49 ^b^^;^^w^	38.15 ± 6.34 ^a^^;^^v^	120.23 ± 9.82 ^a^^;^^v^	1.50 ± 0.48 ^a^^;^^w^	0.00 ± 0.00 ^c^^;^^v^	13.36 ± 0.51 ^c^^;^^v^	185.41 ± 17.29 ^a^^;^^w^^x^	216.50 ± 17.50 ^a^^;^^v^^w^
**Oleaster**	H4	8.80 ± 0.27 ^b^^;^^w^	32.95 ± 1.28 ^a^^;^^v^	110.13 ± 4.98 ^a^^;^^v^	1.59 ± 0.54 ^a^^;^^w^	0.00 ± 0.00 ^c^^;^^v^	9.09 ± 0.18 ^c^^;^^v^	162.56 ± 6.52 ^a^^;^^x^	185.48 ± 5.12 ^a^^;^^w^

Mean± standard deviation. Different letters in the same row indicate significant differences (p <0.05),

^a-c^ refers the varietal effect and

^v-x^ to the Harvest time effect.

Besides, Aglycon forms of ligstroside and oleuropein are the most abundant secoiridoids in the oil samples. According to other researches [41, 45, 46], they are generated by the enzymatic deglycosylation of the ligstroside and oleuropein (abundantly present in the olive) during the oil processing and extraction phases. As for the total phenol, the concentration of individual secoiridoids are influenced by different environmental factors [31, 44]. Thus, oils from Oleaster were characterized by a higher amount of 3,4-DHPEA-EA (28.88–37.52mg/kg), p-HPEA-EA (110.13–124.21mg/kg), total secoiridoids (162.56–185.41mg/kg) and total biophenols (185.48–217.9mg/kg) than the cultivated oils. According to Pérez et al. [46], the genetic component is one of the main factors shaping the phenolic composition of monovarietal olive oil. This result is in agreement with other researches on Algerian varieties [29, 37]. Besides, the oils extracted from Chemlal and Limli cultivars showed high level of elenolic acid, oleocanthal and methyl oleuropein aglycon. The ANOVA test confirmed the significant (P≤ 0.05) influence of the genetic factor on the secoiridoids profile of EVOO. In regard with the ripening process, the majority of individual and total secoiridoids presented a significant and similar decrease (P≤ 0.05), notably by a low levels recorded at the last harvest date. Comparable trends have been observed in others researches [29].

### Antiradical activity and antioxidant capacity

The antioxidant capacity depends on composition and the amount of phenolic and non-phenolic bioactive compounds in the EVOO samples, which are influenced considerably by the change of olive variety and maturation [13, 35]. Hence, high significant differences (P≤0.001) were observed between the varieties (Table 3). According to the results reported by Bouarroudj et al. [29], the Oleaster extracts were distinguished from the cultivated oils by a strong antioxidant capacity, that remains more stable and higher over advanced ripening stage, and confirmed by ratios ranging from 89.93 to 74.66%, 79.84 to 24.17% and 61.95 to 18.39% in Oleaster, Chemlal and Limli oil samples, respectively. Moreover, the antiradical activities decrease significantly (P≤0.001) across the maturation process, and their values registered the fall of more than 30% in DDHP radical scavenging capacity within Chemlal and Limli EVOOs extracts, and almost 18% in Oleaster extracts (vary from 0.503 to 0.419 mg TE/ml) at the final harvest date. In line with other studies on this field [8, 29, 47], the total secoiridoids and biophenols were correlated positively (respectively by r^2^ = 0.777, 0.722, p< 0.05) with the antioxidant activity during the ripening process (S1 Table). Thus, indicate clearly that extracts from the earliest harvest date have the best anti-free radical activity against the DPPH radical. Consumption of oil with higher levels of natural antioxidants is more suitable for limiting the incidence of cardiovascular disease [48].

### PCA results

**Fig 3:**
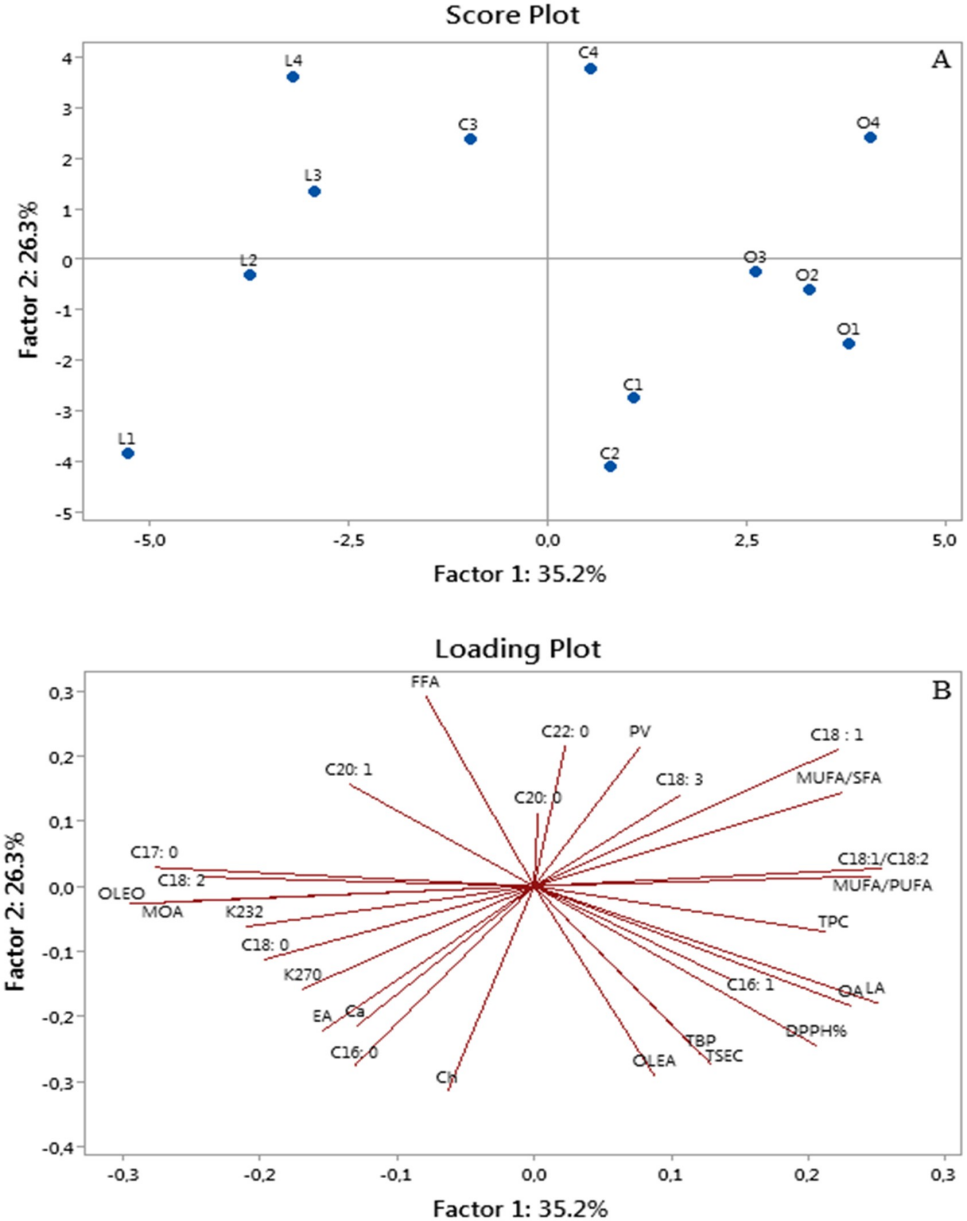
PCA analysis on the most important olive oil parameters. A: Distribution of oil samples based on the variables adopted, B: Vector distribution of variables. Factor 1 and factor 2 explain 61.5% of the total variation. FFA: Free fatty acids, PV: Peroxide value, Ch: Chlorophylls, Ca: Carotenoids, OA: Oleuropein aglycon, LA: Ligstroside aglycon, OLEA: Oleocanthal, OLEA: Oleacein, MOA: Methyl oleuropein aglycon, EA: Elenolic acid, TSEC: Total secoiridoids, TBP: Total Biophenols HPLC. In the score plot graph (B), the letters L, C, and O refer to the varieties Limli, Chemlal, and Oleaster, respectively, and the numbers 1–4 indicate the different harvest dates.

The chemometrics of principal component analysis (PCA) was performed on the most important oil parameters to define the possible interactions between the different EVOO samples and the main correlations among the variables. The PCA results (Fig 3) show that total variance of 61.5% were explained by the first factor (PC1) and the second factor (PC2), which account for 35.2 and 26.3% of variance, respectively.

The variable that more contributes to the first component are C18:1, C18:1/C18:2, MUFA/SFA, MUFA/PUFA, TPC, OA, LA which are correlated positively and K232, C18:2, OLEO, MOA, that associated negatively. Most of the remaining variables, C16:0, Ch, Ca, OLEA, EA, TSC, TBP, DPPH% were negatively correlated with the second component PC2, except for C18:1 and FFA, which were positively related (S2 Table). The score plot presented in Fig 3, segregate the different oils samples, according to the PC1, in two distinct groups. The score graph shows that the Oleaster oils were clustered in the right half of the score plot, distinguished by variables positively correlated with PC1 and also projected in the right side of the loading graph. In other hand, the Limli samples are grouped in the left half of the plot, which are overlapped with the negatives variables (K232, C18:2, OLEO, MOA…) of the loading plot. Besides, Fig 3 showed a non-clear separation for Chemlal samples, with the latter being distributed between Limli and Oleaster groupings. This particular behavior provided by the specific variation in Chemlal oil parameters may be due to the rapid ripening process of the Chemlal fruit (Fig 1), which causes a sharp drop in several oil compounds. Therefore, large gap was observed between the early harvest H1 and H2 samples and those of the H3 and H4 harvests for Chemlal variety in terms of their secoiridoids contents such as oleacein, oleuropein aglycone, and ligstroside aglycone (Table 5) was observed. Another interesting point that might be raised is that monounsaturated fatty acids seem to be responsible for the localisation of Oleaster oils at high PC1 values, whereas saturated fatty acids are particularly relevant in the Limli samples. These observations are in agreement with the fatty acid data in Table 2. Again, the Chemlal samples are clearly different from the other varieties, with the long chain saturated fatty acids being relevant when the late harvest is performed.

In sum, the PC1 was able to allocate the oil samples according to their varietal characteristics. The second largest of variable variation PC2 allowed to separate the oils samples according to their harvest date, therefore it can be seen that the dots C1, C2, L1, O1 representing the early harvest dates are sorted into the lower PCA quadrant, which characterized by high level of total secoiridoids, biophenols, and better antioxidant activity. On the other hand, L3, L4, C3, C4 and O4, which represent the last harvest dates, are projected on the upper quadrant, marked by lower levels in the previous variables. The results obtained from the PCA analysis are in line with the observations previously reported on the parameters under study and offer a more precise view of the contribution of the variety and maturity factors to the characterization and quality of olive oil.

## Conclusion

This investigation on one of the main dietary intakes has led to a better understanding of the effect of changes in varietal source and fruit ripening on the quality of virgin olive oil. The influence of both factors was observed in the considerable differences between the oil samples in terms of oil quality indices (Acidity, Peroxide, K232, and K270), pigment content, fatty acid profile, phenolic content, and antioxidant activity. Thus, the extracts of wild olive oils were characterized by a higher level of bioactive compounds and strong antiradical scavenging capacity, which are probably due to the exceptional adaptation of Oleaster trees to environmental conditions. Moreover, late harvest dates generally have a negative effect on olive oil quality, observed by a significant decrease in chlorophyll, carotenoid, total phenol, secoiridoids content, and antiradical scavenging activity. The variation of antioxidant capacity of the oil samples was strongly correlated with their content on the phenolic compound, particularly the secoiridoids fraction that represents approximately 60–90% of total biophenols. The composition of the latter was dominated by a profile rich in several isomers of oleuropein and ligstroside aglycons, detected by a rapid and efficient method that relies on UHPLC-DAD and the negative electrospray ionisation (ESI) mode of the UHR-Q-TOF mass spectrometer. The aglycon form of oleuropein and ligstroside represent more than 60% of the total secoiridoids in both cultivated and wild varieties. The chemometrics approach of discriminant analysis (PCA) provided a better observation on the variations of the olive oil composition according to the studied factors. According to this analysis, secoiridoids and fatty acid profiles were the most important components contributing to the discrimination between olive oil samples. Further studies are needed for a more precise characterisation of the individual secoiridoids composition of olive oil, including a broader understanding of the process of generation of acetal and hemiacetal forms; elucidation of this mechanism will contribute to better detection and estimation of the content of certain compounds, such as oleocanthal and oleacein.

## Supporting information

S1 Table Pearson correlation matrix of the secoiridoids compounds and inhibition percentage (DPPH%). The correlation test has been carried out with confidence level of 95%. OA: Oleuropein aglycon, LA: Ligstroside aglycon, OLEA: Oleocanthal, OLEA: Oleacein, MOA: Methyl oleuropein aglycon, EA: Elenolic acid, TSEC: Total secoiridoids, TBP: Total Biophenols HPLC.
(DOCX)

S2 Table X-loadings of the different olive oil variables according to principal components 1 and 2. The PCA analysis on 29 parameters of virgin olive oil showed that the first two principal components (PC1 and PC2) were sufficient to display the data structure. PC1 and PC2 account for 35.2 and 26.3% of the variance, respectively.
(DOCX)
