# Supplementary material for: Changes in secoiridoids content and chemical characteristics of cultivated and wild Algerian olive oil, in term of fruit maturation
Source: PLoS One. 2021 Nov 16;16(11):e0260182. doi: 10.1371/journal.pone.0260182 (PMC8594848; doi:10.1371/journal.pone.0260182)
Supplement: S1 Table — The correlation test has been carried out with confidence level of 95%. OA: Oleuropein aglycon, LA: Ligstroside aglycon, OLEA: Oleocanthal, OLEA: Oleacein, MOA: Methyl oleuropein aglycon, EA: Elenolic acid, TSEC: Total secoiridoids, TBP: Total Biophenols HPLC. (DOCX) [file pone.0260182.s001.docx]

**S1 Table.** Pearson correlation matrix of the secoiridoids compounds and inhibition percentage (DPPH%).

|  | DPPH% | OA | LA | OLEO | OLEA | MOA | EA | TSEC |
| --- | --- | --- | --- | --- | --- | --- | --- | --- |
| OA | 0,793 |  |  |  |  |  |  |  |
| LA | 0,869 | 0,933 |  |  |  |  |  |  |
| OLEO | -0,583 | -0,557 | -0,655 |  |  |  |  |  |
| OLEA | 0,653 | 0,656 | 0,612 | -0,099 |  |  |  |  |
| MOA | -0,549 | -0,506 | -0,673 | 0,86 | -0,126 |  |  |  |
| EA | 0,15 | 0,035 | -0,031 | 0,516 | 0,175 | 0,586 |  |  |
| TSEC | 0,777 | 0,877 | 0,84 | -0,227 | 0,714 | -0,176 | 0,445 |  |
| TBP | 0,722 | 0,838 | 0,814 | -0,206 | 0,65 | -0,136 | 0,429 | 0,981 |

The correlation test has been carried out with confidence level of 95%. OA : Oleuropein aglycon, LA : Ligstroside aglycon, OLEA : Oleocanthal, OLEA : Oleacein, MOA: Methyl oleuropein aglycon, EA : Elenolic acid, TSEC : Total secoiridoids, TBP : Total Biophenols HPLC.
