# Supplementary material for: Changes in secoiridoids content and chemical characteristics of cultivated and wild Algerian olive oil, in term of fruit maturation
Source: PLoS One. 2021 Nov 16;16(11):e0260182. doi: 10.1371/journal.pone.0260182 (PMC8594848; doi:10.1371/journal.pone.0260182)
Supplement: S2 Table — The PCA analysis on 29 parameters of virgin olive oil showed that the first two principal components (PC1 and PC2) were sufficient to display the data structure. PC1 and PC2 account for 35.2 and 26.3% of the variance, respectively. (DOCX) [file pone.0260182.s002.docx]

**S2 Table.** X-loadings of the different olive oil variables according to principal components 1 and 2.

|  | **Variable** | **PC1** | **PC2** |
| --- | --- | --- | --- |
| 1 | Acidity | -0,079 | 0,292 |
| 2 | Peroxide value | 0,078 | 0,215 |
| 3 | K232 | -0,211 | -0,063 |
| 4 | K270 | -0,169 | -0,158 |
| 5 | Chlorophylls | -0,063 | -0,316 |
| 6 | Carotenoids | -0,129 | -0,215 |
| 7 | Total phenol | 0,212 | -0,07 |
| 8 | Oleuropein aglycon | 0,232 | -0,185 |
| 9 | Ligstroside aglycon | 0,251 | -0,18 |
| 10 | Oleocanthal | -0,295 | -0,028 |
| 11 | Oleacein | 0,088 | -0,291 |
| 12 | Methyl oleuropein aglycon | -0,271 | -0,025 |
| 13 | Elenolic acid | -0,154 | -0,222 |
| 14 | Total secoiridoids | 0,13 | -0,275 |
| 15 | Total Biophenols HPLC | 0,122 | -0,26 |
| 16 | DPPH% | 0,206 | -0,245 |
| 17 | C16 : 0 | -0,132 | -0,278 |
| 18 | C16 : 1 | 0,144 | -0,144 |
| 19 | C17 : 0 | -0,277 | 0,029 |
| 20 | C18 : 0 | -0,197 | -0,113 |
| 21 | C18 : 1 | 0,223 | 0,211 |
| 22 | C18 : 2 | -0,24 | 0,014 |
| 23 | C18 : 3 | 0,107 | 0,141 |
| 24 | C20 : 0 | 0,003 | 0,113 |
| 25 | C20 : 1 | -0,134 | 0,156 |
| 26 | C22 : 0 | 0,023 | 0,216 |
| 27 | C18:1/C18:2 | 0,254 | 0,027 |
| 28 | MUFA/SFA | 0,224 | 0,144 |
| 29 | MUFA/PUFA | 0,246 | 0,015 |

The PCA analysis on 29 parameters of virgin olive oil showed that the first two principal components (PC1 and PC2) were sufficient to display the data structure. PC1 and PC2 account for 35.2 and 26.3% of the variance, respectively.
